# Annotated Datasets of Oil Palm Fruit Bunch Piles for Ripeness Grading Using Deep Learning

**DOI:** 10.1038/s41597-023-01958-x

**Published:** 2023-02-04

**Authors:** Suharjito, Franz Adeta Junior, Yosua Putra Koeswandy, Debi, Pratiwi Wahyu Nurhayati, Muhammad Asrol, Marimin

**Affiliations:** 1 Industrial Engineering Department, BINUS Graduate Program – Master of Industrial Engineering, Bina Nusantara University, Jakarta, 11480 Indonesia; 2 Computer Science Department, School of Computer Science, Bina Nusantara University, Jakarta, 11480 Indonesia; 3 Computer Science Department, BINUS Online Learning, Bina Nusantara University, Jakarta, 10480 Indonesia; 4 Department of Agro-Industrial Technology, Faculty of Agricultural Engineering and Technology, IPB University (Bogor Agricultural University), Bogor, West Java Indonesia

**Keywords:** Agriculture, Electrical and electronic engineering

## Abstract

The quality of palm oil is strongly influenced by the maturity level of the fruit to be processed into palm oil. Many studies have been carried out for detecting and classifying the maturity level of oil palm fruit to improve the quality with the use of computer vision. However, most of these studies use datasets in the form of images of oil palm fresh fruit bunches (FFB) with incomplete categorization according to real conditions in palm oil mills. Therefore, this study introduces a new complete dataset obtained directly from palm oil mills in the form of videos and images with different categories in accordance with the real conditions faced by the grading section of the palm oil mill. The video dataset consists of 45 videos with a single category of FFB videos and 56 videos with a collection of FFB with multiple categories for each video. Videos are collected using a smart phone with a size of 1280 × 720 pixels with .mp4 format. In addition, this dataset has also been annotated and labelled based on the maturity level of oil palm fruit with 6 categories, which are unripe, under-ripe, ripe, overripe, empty bunches and abnormal fruit.

## Background & Summary

To produce quality palm oil, mature palm fruit is needed. Maturity of Oil Palm Fruit Bunches (FFB) is usually determined by the number of loose fruits falling from the bunch^1^. Besides, maturity can also be seen from the colour of the fruit from black to orange. Usually, determining the maturity of FFB is done by visual inspection of the fruit colour. Visual inspection of colour ripeness has several disadvantages when the FFB is on a tall tree and it depends on the perception of the observer. Detection of ripeness by waiting for fruit to fall can cause crop losses. Detection of ripeness in tall trees makes it difficult for observers to ascertain ripe fruit due to distance and lighting. Many studies related to the detection of oil palm fruit ripeness have been carried out, either with a computer vision approach^2–12^ or with a light sensor approach^13–17^, but they have not obtained satisfactory results because of the complex characteristics of oil palm fruit, such as uneven colour of ripe fruit, oil palm fruit in bunches which looks small, and the different levels of fruit maturity in some varieties. Table 1 shows the results of a study to classify and detect the maturity level of oil palm FFB. The dataset has limitations such as incomplete categorization and a lack of FBB variations, making it substantially different from real-world conditions.

**Table 1:** Existing palm oil FFB studies.

Dataset Name	Number of Classes	Total dataset
Local dataset^2^	3 classes (raw, under-ripe, and ripe	160 (images)
Central Kalimantan^3^	6 classes (unripe, under-ripe, and ripe, overripe, abnormal, empty)	653 (images)
Felda Agricultural Services Sdn. Bhd (FASSB)^4^	4 classes (unripe, under-ripe, ripe and overripe.	80 FFBs were 208 (images)
palm oil estate in Johor^5^	2 classes (ripe and unripe)	264 (images)
Local dataset from Indonesia^6^	7 classes (Ripening, Raw, less Ripped, Almost Ripped, Ripped, Perfectly ripped, too ripped)	400 (images)
Local dataset from Malaysia^8^	4 classes (unripe, under-ripe, ripe and overripe)	120 (images)
United Plantation Research and Development in Teluk Intan (UPRD), Perak, Malaysia^10^	3 categories (under-ripe, ripe, and over-ripe)	297 oil palm FFBs (images)
Local dataset from Malaysia^11^	2 categories (ripe, unripe)	490 FFBs (images)
Local dataset from Malaysia^13^	3 categories (under-ripe, ripe and overripe)	120 oil palm FFBs (images)
Local dataset from West Java^15^	3 categories (under-ripe, ripe and overripe)	180 FFBs (images)

**Fig. 1:**
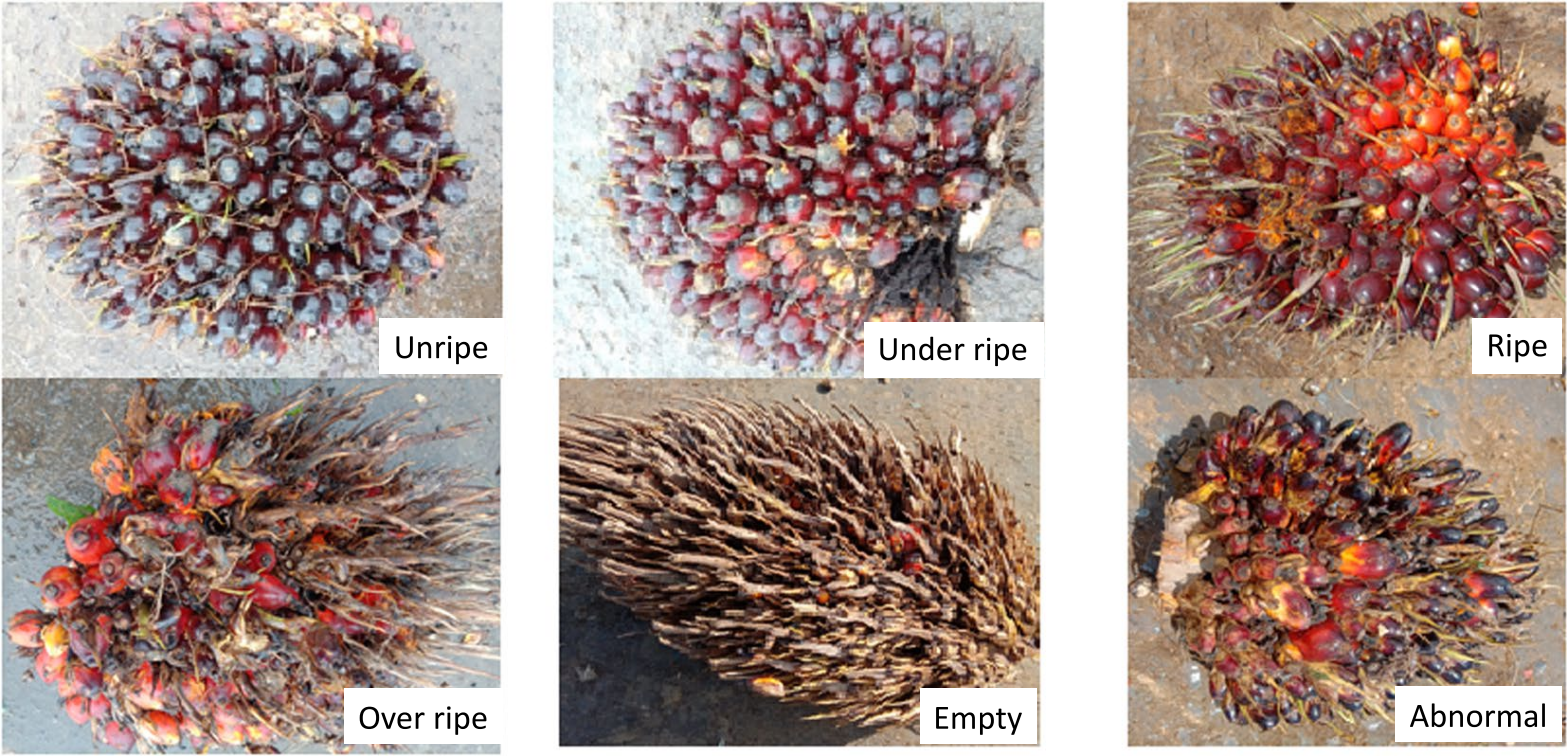
Example images of the maturity level of oil palm FFB.

Research using computer vision is usually done based on the input image to detect the colour of the fruit, while research with a light sensor is done by analysing the results of the spectrum of light emitted to the oil palm fruit. Most previous studies used oil palm image input or the colour spectrum of oil palm fruit because with this input the detection process is more efficient. Several previous studies using a computer vision approach with an input image have been carried out by using the SVM method with 3 classes^18^, namely raw, under-ripe and ripe. Research with deep learning for ripeness detection has been carried out by using EfficientNet^3^ with single image datasets. Real time oil palm ripeness detection using YOLOv4 with 3 classes dataset has been proposed^19^ for harvesting system and another research of real time ripeness detection at harvesting process has been proposed using YOLOv3^20^. Based on the results of this literature study, there are no oil palm datasets in the form of images or videos of collections or piles of oil palm fresh fruit bunches with various categories or single categories. This paper provides image and video datasets from collections or piles of oil palm fresh fruit bunches taken directly from palm oil mills in South Kalimantan. In the grading section, smart phones were used with 6 levels of oil palm fruit maturity levels, which are unripe, under-ripe, ripe, overripe, empty bunches and abnormal fruit (Fig. 1). There is research to detect oil palm in real-time using YOLOv4, the data used is fresh fruit bunches of oil palm that are still attached to trees with ripe and unripe classes^11^. However, this research is not fully applicable because it can only be used on oil palm plantations, whereas to conduct an assessment at a palm oil mill requires more than 2 classes to avoid inappropriate maturity levels.

**Fig. 2:**
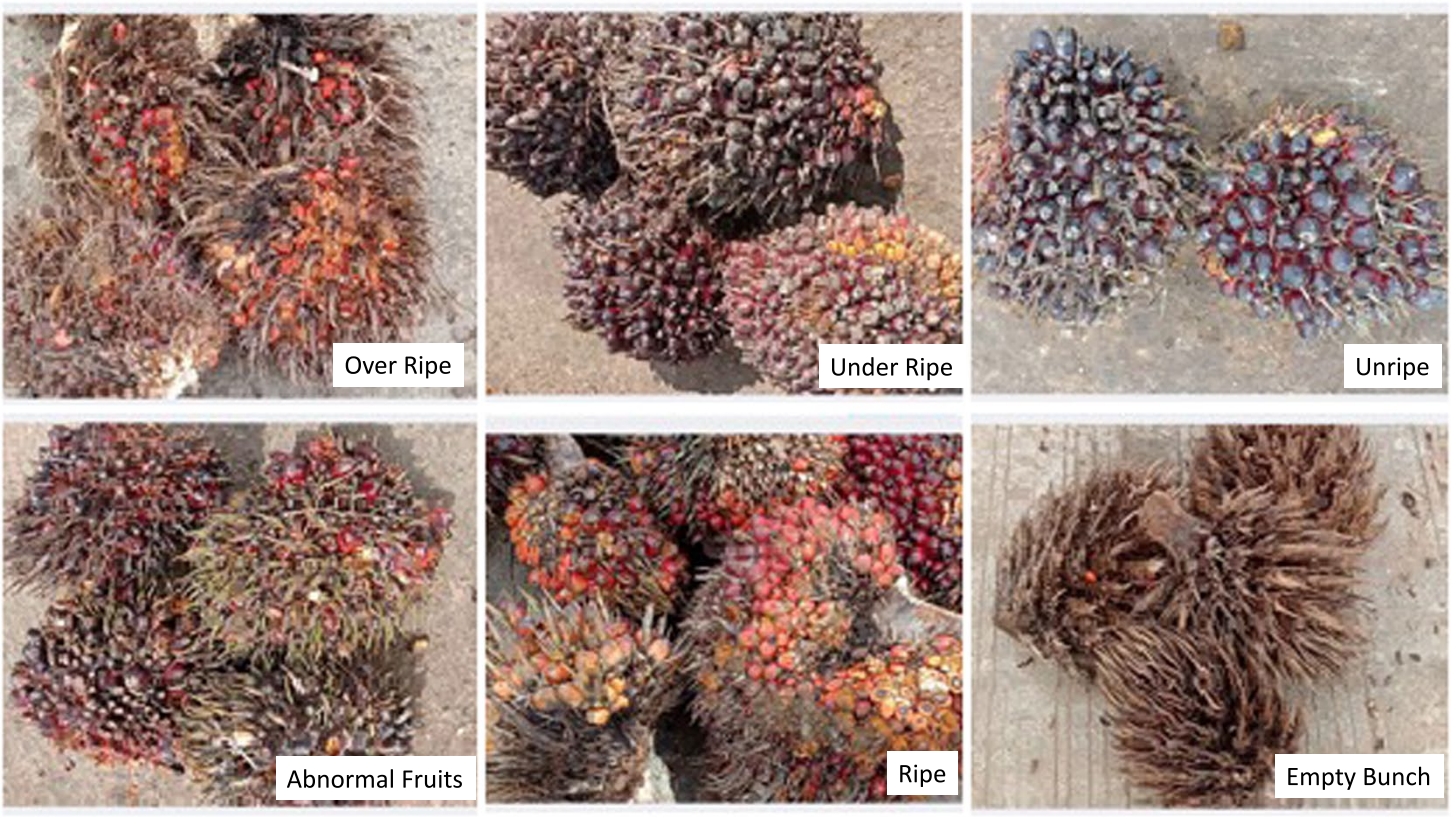
Example of oil palm FFB piles for single category per image.

**Fig. 3:**
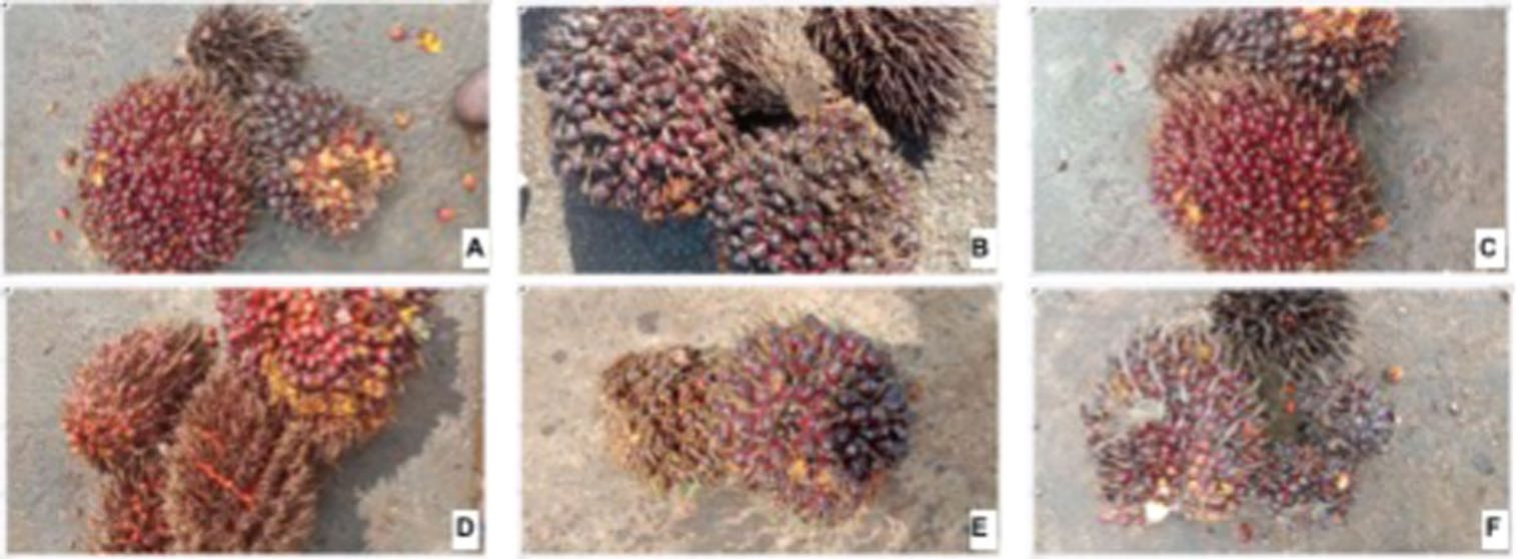
Examples of oil palm fresh fruit bunches piles for multi categories per image. (**A**) 1 empty, 1 ripe, and 1 unripe; (**B**) 2 empty and 2 unripe; (**C**) 1 empty, 1 ripe, and 1 unripe; (**D**) 2 ripe and 2 overripe; (**E**) 1 under-ripe and abnormal; (**F**) 1 ripe, 1 empty, and 2 abnormal fruits.

This dataset is multimodal data in the form of videos and images of oil palm fresh fruit bunches with 6 categories that have been determined and validated by experts in grading the level of maturity of oil palm fruit in palm oil mills. This dataset can be used by many stakeholders such as students and researchers, application developers, machine learning and deep learning engineers, data scientists, agronomists and palm oil mill graders and other researchers. Datasets are very useful for application developers to test data and develop machine learning models that can be used to create smartphone-based applications or applications embedded in robots or other devices. This data is also very useful for data scientists to be able to find the right method for classifying and detecting fruit ripeness. Besides, this data is also very useful for developing deep learning algorithms to classify and detect ripeness as well as fruit counting effectively and efficiently. In the real world application, consistency is required in assessing the maturity level of oil palm so that there are no errors in estimating the maturity level and causing losses to the palm oil processing mill. Some video data properties, such as dynamic luminance, objects partially obscured by other objects, and motion blurs when transitioning between frames, are identical to how the human eye works^21^ so that the video (or sequential image) will be more applicable in the real world compared to image pieces that have no connection between frames. This dataset is a collection of videos on the maturity level of oil palm fruit with a single category for each video and with multiple categories for each video. An example of a dataset with a single category for each video can be seen in Fig. 2, while an example of data with multiple categories for each image can be seen in Fig. 3. By using a combination of the multiple categories, the dataset can produce machine learning that is in accordance with real conditions in the field so that better model performance can be obtained compared to using datasets with a single category.

## Methods

**Fig. 4:**
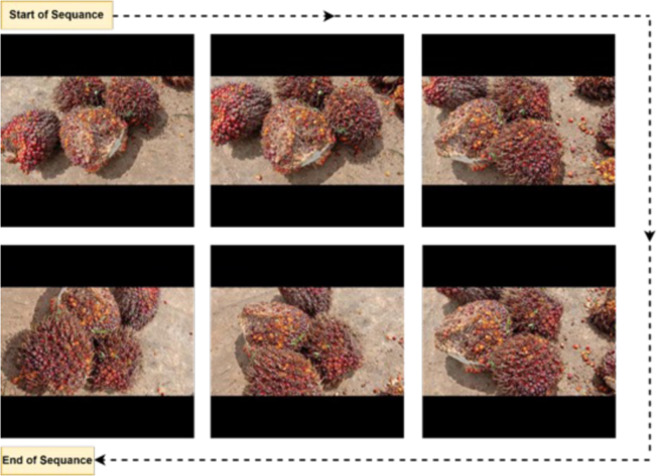
Example of a video recording frame obtained using the 360° method. Various FFB positions can be captured to show the condition of the oil palm FFB maturity level.

The dataset was collected from some palm oil mills in the section on grading the maturity level of oil palm fruit in South Kalimantan, Indonesia. Oil palm fresh fruit bunches with various levels of maturity were collected and recorded using a smartphone on the concrete cement floor background at factory backyard. The recording strategy uses rotating the camera 360° around the pile of palm oil FFB to capture the most of the FFB positions. A variation of the position of the FFB can be obtained by rotating 360° and can represent the whole condition of the oil palm’s maturity level, an example of the position variation in the FFB can be seen in Fig. 4. The video was captured throughout the day, between 12.00 and 13.00 p.m., in sunny weather conditions. Due to weather issues that are not always sunny, the total time required to gather the dataset is estimated to be two months. So, there are several different variations of oil palm FFB obtained.

**Fig. 5:**
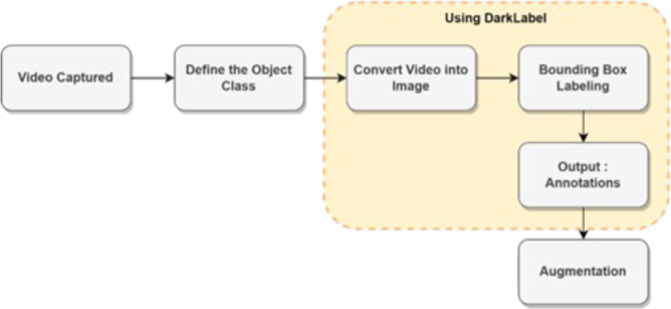
Illustration of Data pre-processing stages.

Figure 5 is a pre-processing flow that is carried out to process raw data into ready-to-use data. The raw data used is in mp4 format with a resolution of 1280 × 720 and taken using a smartphone. All types of object classes have been determined and evaluated by palm oil experts at the palm oil grading site. The data that can be used to conduct training on the deep learning model is in the form of images. Therefore, we extracted the frames on the video into sequential images. Frame extraction was carried out with the VLC Media Player^22^ application with a recording ratio configuration of 30. It aims to extract 1 frame every 1 second so that the possibility of image redundancy is very small^21^. The resulting output resolution is 416 × 416.

**Fig. 6:**
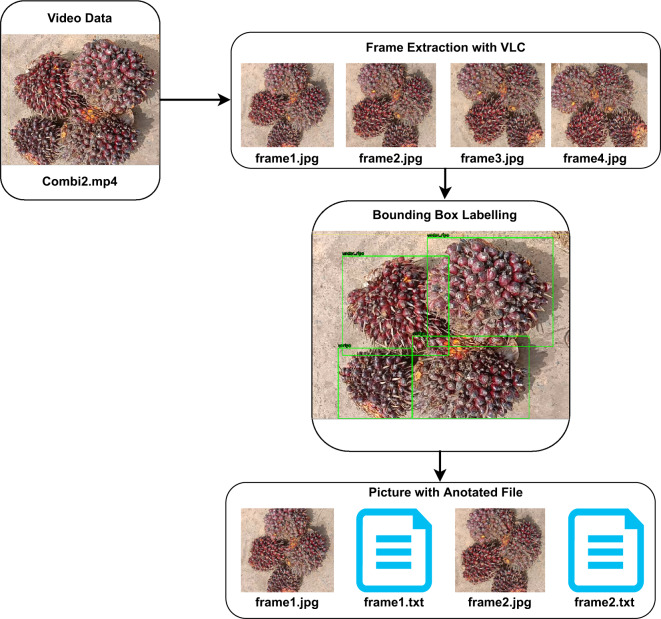
The illustration of Oil Palm FFB Video’s labelling and annotation.

Sequential images with a resolution of 416 × 416 successfully extracted from the video were given a bounding box. The process of giving bounding boxes was done using DarkLabel^23^. DarkLabel is a tool for annotating object detection, annotation formats available in DarkLabel are Pascal VOC, YOLO, and Multiple Object Tracking (MOT). In pre-processing stage, bounding boxes were assigned manually to each image to ensure the density between the box and the object, the illustration of bounding box annotator can be seen in Fig. 6. The annotation format is stored in the form of a YOLO annotation (.txt) consisting of [class id, x, y, w, h] where x and y are the coordinates of the box, w is the width, and h is the height. The results of each class that has been given a bounding box are stored in a different file. Bounding box is given by making a box-shaped barrier. The box shape is rearranged so that the boundary surrounds the object you want to detect Annotation file has the same name as the annotated image name and placed in the same folder.

## Data Records

Based on Fig. 6, data was recorded in two modals namely videos dataset and image dataset. Video datasets contain 45 file of single category and 56 file of multi categories oil palm FFB. Image datasets have been annotated using Roboflow^24^ software than can be used as input data for ripeness detection and classification using YOLO model. The datasets is available at Science Data Bank^25^. The video data criteria used were: (1) recordings with 360° rotation of oil palm FFB and (2) video duration of approximately 10 to 15 seconds. Then based on the video criteria used, 1 frame is extracted for every second. Based on Table 2, the total extracted images from video of oil palm FFB file are 4160 files with 14559 objects and 7171 image. The total files of images of each maturity category of oil palm FFB were different from the sum of images, because each image file has more than one object class in the piles of oil palm FFB. The datasets have been split into data training, validation, and testing using composition 70:20:10 with the total images are 2908 for training, 835 for validation and 417 for testing. The detail of image and object for each category can be seen in Table 2.

**Table 2:** The distribution of image and Object for each class category.

Category of FFB	#Images	#Objects
Unripe	1130	2913
Under-ripe	1289	2575
Ripe	1880	2974
Over Ripe	1162	2641
Empty Bunch	473	857
Abnormal FFB	1237	2599
Total	7171	14559

## Technical Validation

**Fig. 7:**
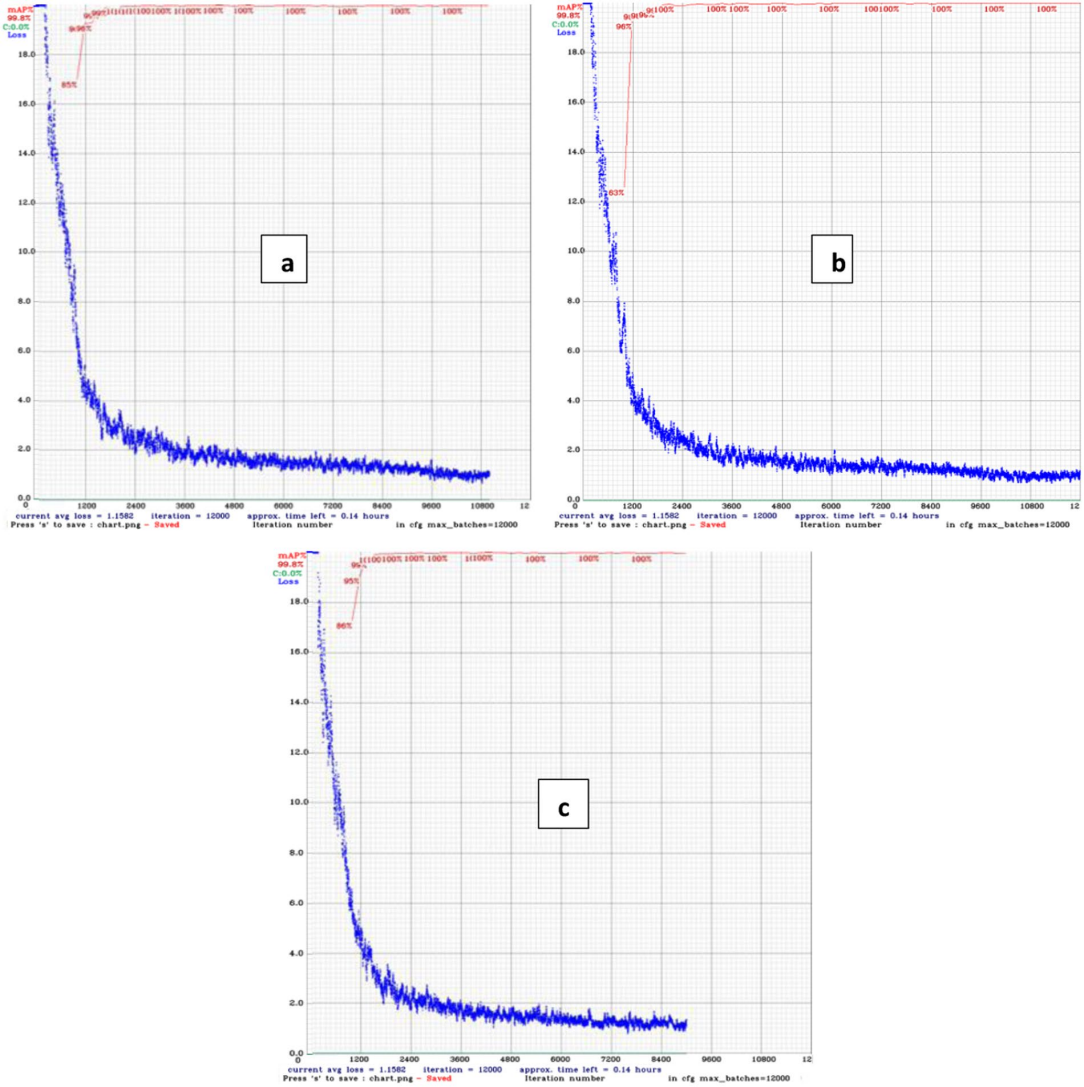
Training loss & validation mAp chart of the models (**a**) YOLOv4-320, (**b**) YOLOv4-416 and (**c**) YOLOv4-512.

**Fig. 8:**
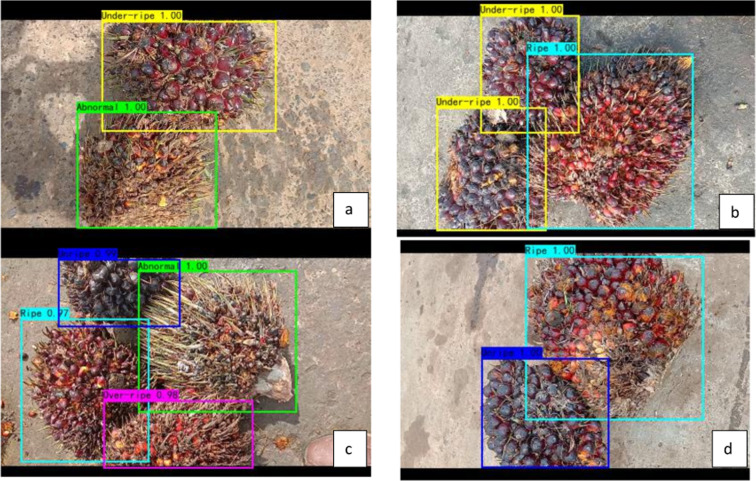
Testing result example of YOLOv4 Models for detection and classification of palm oil ripeness with video input; (**a**) Un-ripe & Abnormal FFB; (**b**) 2 Under-ripe and 1 Ripe FFB; (**c**) Unripe, Abnormal, Ripe and over-ripe FFB; (**d**) Unripe and ripe FFB.

For data validation, it was tested using the YOLOv4 models^22^ with hyper-parameters as shown in Table 3. To suit the dataset, hyper-parameter values such as width, height, max batches, and steps are modified. This change was implemented in accordance to recommendations from the initial YOLOv4 research^26^. Figure 7 shows a graph of the model’s performance during training and validation. Based on the graph indicates the performance of validation loss was convergence to zero and based on the value of mAP that closed to 1 indicates that the models have good performance. Table 4 presents the test result of each YOLO model used. Figure 8 shows testing result of the of the model with input video of palm oil FFB with multi category of ripeness. The data utilized for training, validation, and testing is composed of consecutive images successfully retrieved from video, making them more applicable to real-world applications. The sequential image structure also enables the model to determine the FFB’s development level from multiple perspectives.

**Table 3:** Hyper-parameter of YOLO4 for data validation.

	YOLOv4-320	YOLOv4-416	YOLOv4-512
Batch	64	64	64
Subdivisions	16	16	16
Width	320	416	512
Height	320	416	512
Channels	3	3	3
Momentum	0.949	0.949	0.949
Decay	0.0005	0.0005	0.0005
Learning rate	0.001	0.001	0.001
Burn in	1000	1000	1000
Max batches	12000	12000	12000
Steps	9600, 10800	9600, 10800	9600, 10800
Policy	Steps	Steps	Steps
Scales	.1, .1	.1, .1	.1, .1

**Table 4:** Testing Results of YOLO4 models.

Model	Precision	Recall	F1-score	Avg IoU	TP	FP	FN
YOLOv4-320	0.99	1.00	0.99	88.01%	1406	20	4
YOLOv4-416	0.97	1.00	0.98	88.27%	1404	37	6
YOLOv4-512	0.99	1.00	0.99	88.94%	1408	20	2

Unfortunately, the open datasets used in the current study on oil palm ripeness are not available. Comparatively, the current research’s typical dataset attempts to increase the grade and output of refined palm oil^2–6,8,10,11,13,15^. The video dataset employed in this study, however, is concentrated on offering an assessment of the oil palm FFB maturity level, especially in oil palm processing facilities. Video can be used as a dataset since it closely reflects real-time situations, which makes it more appropriate for real-time grading procedures. The use of a video dataset and a real-time object detection algorithm can improve the speed of determining the oil palm FFB’s maturity level. However, using video datasets presents several challenges. Compared to using non-sequential photos, pre-processing will be more difficult. Then the data used in carrying out the training process will become more numerous so that it can make the model training time longer. In addition, the background contained in this dataset is the backyard of the place for grading the level of maturity of the oil palm so that the results of direct detection on oil palm plantations may experience a decrease in performance because the background on the oil palm plantation is more complex than the background from the factory backyard where the FFB of palm oil ripeness is graded.

## Usage Notes

The existing dataset has some limitations as follows:

1. The dataset consists of image classes that are not balanced for each category due to the availability of data in the grading process to obtain abnormal data and empty bunches are difficult to obtain from shipping to palm oil mills.
2. The dataset has not been augmented so that to get better performance in the model development, it is necessary to do data augmentation in order to increase the dataset.

## Data Availability

The software used to process the dataset consists of software for converting video data into a collection of images using VLC (https://www.videolan.org/index.id.html). Software used for labelling and making image bounding boxes using Darklabel is provided by https://github.com/darkpgmr/DarkLabel. Software used for converting labelled data into data that is ready for input in processed modelling with a deep learning is the Roboflow (https://roboflow.com/) and the software used for data validation is the Python program.

## References

[1] Chew, C. L. *et al*. Exogenous ethylene application on postharvest oil palm fruit bunches improves crude palm oil quality. *Food Sci. Nutr*. **9** (2021). 10.1002/fsn3.2423 PMC8498053 34646505

[2] Septiarini, A., Hamdani, H., Hatta, H. R. & Anwar, K. Automatic image segmentation of oil palm fruits by applying the contour-based approach. *Sci. Hortic. (Amsterdam)*. **261** (2020).

[3] Suharjito, Elwirehardja GN, Prayoga JS (2021). Oil palm fresh fruit bunch ripeness classification on mobile devices using deep learning approaches. Comput. Electron. Agric..

[4] Fadilah, N., Mohamad-Saleh, J., Halim, Z. A., Ibrahim, H. & Ali, S. S. S. Intelligent color vision system for ripeness classification of oil palm fresh fruit bunch. *Sensors (Switzerland)* **12** (2012). 10.3390/s121014179 PMC3545614 23202043

[5] Sabri, N., Ibrahim, Z., Syahlan, S., Jamil, N. & Mangshor, N. N. A. Palm oil fresh fruit bunch ripeness grading identification using color features. *J. Fundam. Appl. Sci*. **9** (2018).

[6] Pamornnak, B., Limsiroratana, S. & Chongcheawchamnan, M. Oil content determination scheme of postharvest oil palm for mobile devices. *Biosyst. Eng*. **134** (2015).

[7] Herman, H., Susanto, A., Cenggoro, T. W., Suharjito, S. & Pardamean, B. Oil Palm Fruit Image Ripeness Classification with Computer Vision using Deep Learning and Visual Attention. *Journal of Telecommunication*, *Electronic and Computer Engineering (JTEC)* vol. 12 (2020).

[8] Ibrahim, Z., Sabri, N. & Isa, D. Palm oil fresh fruit bunch ripeness grading recognition using convolutional neural network. *J. Telecommun. Electron. Comput. Eng*. **10** (2018).

[9] Ishak, W. I. W. & Hudzari, R. M. Image based modeling for oil palm fruit maturity prediction. *J. Food, Agric. Environ*. **8** (2010).

[10] Zolfagharnassab S, Shariff ARBM, Ehsani R, Jaafar HZ, Aris I (2022). Bin. Classification of Oil Palm Fresh Fruit Bunches Based on Their Maturity Using Thermal Imaging Technique. Agriculture.

[11] Lai JW, Ramli HR, Ismail LI, Hasan WZW (2022). Real-Time Detection of Ripe Oil Palm Fresh Fruit Bunch Based on YOLOv4. IEEE Access.

[12] Ghazali, S. A., Selamat, H., Omar, Z. & Yusof, R. Image Analysis Techniques for Ripeness Detection of Palm Oil Fresh Fruit Bunches. *Elektr. J. Electr. Eng*. **18** (2019).

[13] Saeed, O. M. B. *et al*. Classification of oil palm fresh fruit bunches based on their maturity using portable four-band sensor system. *Comput. Electron. Agric*. **82** (2012).

[14] Huddin A (2018). A Rapid and Non-Destructive Technique in Determining The Ripeness of Oil Palm Fresh Fruit Bunch (FFB). J. Kejuruter..

[15] Makky, M. A Portable Low-cost Non-destructive Ripeness Inspection for Oil Palm FFB. *Agric. Agric. Sci. Procedia* **9** (2016).

[16] Makky, M. & Soni, P. *In situ* quality assessment of intact oil palm fresh fruit bunches using rapid portable non-contact and non-destructive approach. *J. Food Eng*. **120** (2014).

[17] Makky, M., Soni, P. & Salokhe, V. M. Automatic non-destructive quality inspection system for oil palm fruits. *Int. Agrophysics* **28** (2014).

[18] Septiarini, A., Hamdani, H., Hatta, H. R. & Kasim, A. A. Image-based processing for ripeness classification of oil palm fruit. *Proceeding - 2019 5th Int. Conf. Sci. Inf. Technol. Embrac. Ind. 4.0 Towar. Innov. Cyber Phys. Syst. ICSITech 2019* 23–26, 10.1109/ICSITech46713.2019.8987575 (2019).

[19] Junos MH, Mohd Khairuddin AS, Thannirmalai S, Dahari M (2021). An optimized YOLO-based object detection model for crop harvesting system. IET Image Process..

[20] Mohd Basir Selvam, N. A., Ahmad, Z. & Mohtar, I. A. Real Time Ripe Palm Oil Bunch Detection using YOLO V3 Algorithm. In *19th IEEE Student Conference on Research and Development: Sustainable Engineering and Technology towards Industry Revolution, SCOReD 2021*, 10.1109/SCOReD53546.2021.9652752 (2021).

[21] Wang J, Zhang T, Cheng Y, Al-Nabhan N (2021). New Generation Deep learning for Video Object Detection: A survey. Comput. Syst. Sci. Eng..

[22] Parico AIB, Ahamed T (2021). Real Time Pear Fruit Detection and Counting Using YOLOv4 Models and Deep SORT. Sensors (Switzerland).

[23] Habtemariam LW, Zewde ET, Simegn GL (2022). Cervix Type and Cervical Cancer Classification System Using Deep Learning Techniques. Med. Devices Evid. Res..

[24] Syaefudin, A., Setiawan, W., Widiatmoko, F., Sofyan, E. & Restu, R. N. Computer Vision with Deep Convolutional Neural Network Approach for Cold-Flow Casting Defect Detection. In *Conference on Management and Engineering in Industry (CMEI)* 31–36, 10.33555/cmei.v3i5.111 (2021).

[25] Suharjito & Junior FA (2022). Annotated Video Dataset of Oil Palm Fruit Bunch Piles for Ripeness Grading[DS/OL]. Science Data Bank.

[26] Bochkovskiy, A., Wang, C.-Y. & Liao, H.-Y. M. YOLOv4: Optimal Speed and Accuracy of Object Detection. (2020).

